# The Subcellular Distribution of Alpha-Tocopherol in the Adult Primate Brain and Its Relationship with Membrane Arachidonic Acid and Its Oxidation Products

**DOI:** 10.3390/antiox6040097

**Published:** 2017-11-26

**Authors:** Emily S. Mohn, Matthew J. Kuchan, John W. Erdman, Martha Neuringer, Nirupa R. Matthan, Chung-Yen Oliver Chen, Elizabeth J. Johnson

**Affiliations:** 1 Jean Mayer US Department of Agriculture Human Nutrition Research Center on Aging, Tufts University, Boston, MA 02111, USA; Nirupa.Matthan@tufts.edu (N.R.M.); Oliver.Chen@tufts.edu (C.-Y.O.C.); Elizabeth.Johnson@tufts.edu (E.J.J.); 2 Discovery Research, Abbott Nutrition, Columbus, OH 43229, USA; Matthew.Kuchan@abbott.com; 3 Department of Food Science and Human Nutrition, University of Illinois at Urbana-Champaign, Urbana, IL 61802, USA; jwerdman@illinois.edu; 4 Oregon National Primate Research Center, Oregon Health and Science University, Beaverton, OR 97006, USA; neuringe@ohsu.edu

**Keywords:** α-tocopherol, brain, membranes, arachidonic acid, isoprostanes, rhesus monkey

## Abstract

The relationship between α-tocopherol, a known antioxidant, and polyunsaturated fatty acid (PUFA) oxidation, has not been directly investigated in the primate brain. This study characterized the membrane distribution of α-tocopherol in brain regions and investigated the association between membrane α-tocopherol and PUFA content, as well as brain PUFA oxidation products. Nuclear, myelin, mitochondrial, and neuronal membranes were isolated using a density gradient from the prefrontal cortex (PFC), cerebellum (CER), striatum (ST), and hippocampus (HC) of adult rhesus monkeys (*n* = 9), fed a stock diet containing vitamin E (α-, γ-tocopherol intake: ~0.7 µmol/kg body weight/day, ~5 µmol/kg body weight/day, respectively). α-tocopherol, PUFAs, and PUFA oxidation products were measured using high performance liquid chromatography (HPLC), gas chromatography (GC) and liquid chromatography-gas chromatography/mass spectrometry (LC-GC/MS) respectively. α-Tocopherol (ng/mg protein) was highest in nuclear membranes (*p* < 0.05) for all regions except HC. In PFC and ST, arachidonic acid (AA, µg/mg protein) had a similar membrane distribution to α-tocopherol. Total α-tocopherol concentrations were inversely associated with AA oxidation products (isoprostanes) (*p* < 0.05), but not docosahexaenoic acid oxidation products (neuroprostanes). This study reports novel data on α-tocopherol accumulation in primate brain regions and membranes and provides evidence that α-tocopherol and AA are similarly distributed in PFC and ST membranes, which may reflect a protective effect of α-tocopherol against AA oxidation.

## 1. Introduction

Vitamin E is an essential, fat-soluble nutrient, obtained from nuts, oils, and green leafy vegetables [1]. Vitamin E has eight structural isomers: 4 (α-, β-, γ- and δ-)tocopherols and 4 (α-, β-, γ- and δ-)tocotrienols; however, mammals preferentially take up and use α-tocopherol [2,3]. α-Tocopherol is crucial for proper brain function, and deficiency can result in a number of neurologic symptoms, such as ataxia, peripheral neuropathy, myopathy, and retinopathy, which can be reversed with supplementation [1]. Although deficiency is rare, there is considerable evidence linking lower α-tocopherol intake levels with cognitive decline and neurodegenerative disease. Epidemiological studies have reported that lower tocopherol intake is associated with poorer cognitive function, compared to individuals with higher intakes [4], and reduced levels of α-tocopherol in plasma [5,6] and cerebrospinal fluid [7,8] have been reported in patients with mild cognitive impairment and Alzheimer’s Disease (AD), the most common form of dementia. Post-mortem brain concentrations of α-tocopherol have also been shown to be positively related to cognitive test scores in a centenarian population [6].

The brain is one of the most metabolically active organs in the body, which causes the production of reactive oxygen species (ROS). As a result, it is highly susceptible to ROS attacks on lipid, protein, and DNA [9]. This susceptibility is accentuated by the high concentrations of polyunsaturated fatty acids (PUFA) found in brain cell membranes. Enhanced oxidative stress is thought to contribute to the pathogenesis of cognitive impairment and dementia [10]. α-Tocopherol’s function as a lipid antioxidant may underlie its association with cognition. Therefore, it is possible that α-tocopherol maintains the integrity of cell membranes through inhibiting oxidation of PUFAs [11], particularly arachidonic acid (AA) and docosahexaenoic acid (DHA), the two major PUFAs found in the brain [12], and thereby combats the cell damage and neurodegeneration underlying cognitive impairment. In addition, emerging evidence indicates this vitamin may also possess non-antioxidant functions, including the regulation of gene expression [13,14]. In vitro and ex vivo studies have reported that treatment with α-tocopherol can down-regulate the expression of pro-inflammatory genes [15,16] and modulate the expression of several cell signaling and cell cycle genes [15,16,17,18]. Thus, the mechanism of action underlying the inverse association between α-tocopherol and cognitive impairment/dementia in humans is of considerable interest.

It is well established that α-tocopherol accumulates within membranes [19], and its subcellular distribution has been investigated in rat brains [20,21]. However, its distribution among different types of membranes and its direct association with membrane PUFA content and brain PUFA oxidation products have not been determined in primates. Determining the subcellular localization of α-tocopherol in brain tissue may provide insight into understanding its potential function(s). The objective of the present study was to determine the distribution of α-tocopherol in membranes from different brain regions and to characterize the relationship between membrane-specific α-tocopherol, membrane PUFA content, and brain PUFA oxidation products (isoprostanes [IsoP] and neuroprostanes [NP]) in primates. This study was performed in rhesus monkeys because they are a well-accepted model for human brain physiology [22].

## 2. Materials and Methods

### 2.1. Animals and Diet

Rhesus monkey (*Macaca mulatta*) brains, analyzed in the present study, were from a subgroup of a larger parent study sample, which investigated the effect of lutein supplementation on lutein accumulation in brain regions and membranes of primates [23]. Our study samples included serum and brain tissue from 6 female and 3 male adult monkeys (mean age: 11.7 ± 3.3 years; mean body weight: 7.87 ± 2.24 kg) that were obtained through the Oregon National Primate Research Center (ONPRC) (Beaverton, Oregon) Tissue Distribution Program; animals were not euthanized specifically for this study, but were euthanized for other projects or for veterinary reasons. All animals had consumed a standard stock diet (Monkey Diet Jumbo 5037, Lab Diet, St. Louis, MO, USA) at least twice a day, along with daily supplements of a variety of fruits and vegetables. The stock diet was measured for vitamin E (α- and γ-tocopherol), using previously reported methods [24]. The n-6 PUFA content of the diet was predominantly linoleic acid (1.7% of ration), with less than 0.01% of each ration being arachidonic acid (AA). The n-3 PUFA content was 0.13% of ration, mainly contributed by linolenic acid (0.10% of ration), but also containing DHA (~0.01% of ration). Our previous study [23] also included tissue from 4 monkeys receiving lutein supplementation, but these animals were not included in the current study, due to potential confounding effects on measures of oxidation.

The study was in compliance with all institutional and federal regulations on the use of laboratory animals as well as the Guide for the Care and Use of Laboratory Animals [25]. Procedures were approved by the Institutional Animal Care and Use Committee (IACUC) of Oregon Health and Science University (Protocol IS00003766). Professional care was provided by the ONPRC Division of Comparative Medicine and all animals were observed at least twice a day by trained veterinary technicians. In addition to rotating dietary supplements of fruits and vegetables, animals were provided with environmental enrichments, including a changing variety of toys. Euthanasia was conducted by a veterinary pathologist; animals were sedated with ketamine, and then deeply anesthetized with sodium pentobarbital, according to the Guidelines of the American Veterinary Medical Association. IACUC approval from Tufts University was also obtained for biological sample receipt, storage, and analysis.

### 2.2. Serum and Brain Collection

Fasting blood was drawn from the saphenous vein at the time of euthanasia. Because the monkeys were not euthanized exclusively for this study, blood samples were not collected in every case but were available for 7 out of 9 animals. Blood was processed for serum (1000× *g*, 10 min, 4 °C) and then stored at −80 °C prior to analysis.

Brains were removed immediately after euthanasia and the prefrontal cortex (PFC), cerebellum (CER), striatum (ST), and hippocampus (HC) were dissected from the right and left hemispheres. The regions consisted of both gray and white matter but excluded major white matter tracts. The dissected regions were immediately placed on dry ice and then stored at −80 °C. The right and left hemispheres of each region were pooled, pulverized in liquid nitrogen, aliquoted, and stored at −80 °C.

### 2.3. Preparation of Brain Membranes

Nuclear, myelin, mitochondrial, and neuronal plasma membranes were isolated from the PFC, CER, ST, and HC, using differential centrifugation with a Ficoll density gradient [26,27]. Pulverized brain samples were homogenized in aqueous buffer (10 mM hydroxyethyl piperazineethanesulfonic acid (HEPES), 0.25 mM ethylenediaminetetraacetic acid EDTA, 0.32 M sucrose, pH 7.2), containing protease inhibitors (cOmpleteTM protease inhibitor cocktail, Roche, Basel, Switzerland). The homogenates were subsequently subjected to low-speed centrifugation (1000× *g*, 4 °C) to isolate the crude nuclear membrane pellet. The resulting supernatant was removed to a new tube and the protocol was repeated with the pellet. Supernatant from the second low-speed centrifugation was combined with the first. The combined supernatants were then centrifuged (17,000× *g*, 4 °C) to obtain the “crude membrane pellet”, containing myelin, mitochondrial, and neuronal plasma membranes. The crude membrane pellet was re-homogenized in buffer containing protease inhibitors but no sucrose (10 mM HEPES, 0.25 mM EDTA, pH 7.2) and was applied to a Ficoll density gradient (consisting of 14% and 7% Ficoll solutions). The homogenate was then subjected to high-speed centrifugation (87,000× *g*, 4 °C), to separate myelin, mitochondrial, and neuronal plasma membranes. All membranes, including the crude nuclear membrane, were purified via centrifugation at 17,000× *g*, 4 °C. Pure membranes were aliquoted and stored at −80 °C for tocopherol and fatty acid analyses. Membrane recovery was 76 ± 1%, as determined by measuring the sum of α-tocopherol levels in all membranes and supernatants and comparing this to total α-tocopherol in each brain region.

### 2.4. α-Tocopherol Extraction from Brain Regions, Membranes, and Serum

The process for the extraction of α- and γ-tocopherol from brain regions and membranes was adapted from Park et al. [28] and has been previously described in detail [29]. Briefly, after the additions of an internal standard (echinenone) and antioxidant (sodium ascorbate), samples were saponified with 5% sodium hydroxide and α-tocopherol was extracted using hexane. Extracts were then injected into a reverse-phase HPLC system to separate and quantify α- and γ-tocopherol. 

Both α- and γ-tocopherol were extracted and analyzed from serum, using a previously published Folch method [30]. The lower limit of detection was 2.7 pmol, and the interassay coefficient of variation (CV) was 4%. Concentrations are expressed as ng/mg protein.

### 2.5. Fatty Acid Extraction and Protein Determination in Brain Regions and Membranes

Lipids were extracted overnight from homogenates of brain regions and membranes using a modified Folch method [31]. Fatty acids were analyzed using an established gas chromatography method [32]. Peaks of interest were identified by comparison with authentic fatty acid standards (Nu-Chek Prep, Inc., Elysian, MN, USA) and expressed as µg/mg protein and mole percent (%). The interassay CV ranged from 0.5 to 4.3% for fatty acids present at levels >5% of total fatty acids (TFAs), 1.8–10.1% for fatty acids present at levels between 1–5% TFAs, and 9.8–25.1% for fatty acids present at levels <1% TFAs. For the present analysis, we focused on the content of total PUFA, total n-6, total n-3, AA, and DHA.

Delipidated brain tissue and membranes from the overnight lipid extraction were digested in 1N sodium hydroxide for the determination of protein, using the bicinchoninic acid (BCA) assay, as per the manufacturer’s instructions (Pierce Inc., Rockford, IL, USA). Brain regions and membranes were digested for 8 and 5 days, respectively.

### 2.6. PUFA Oxidation Determination in Brain Tissue

Total NP and IsoP, formed from the oxidation of DHA and AA, respectively, were extracted from the PFC, CER and ST, and quantified using published methods [33,34] with modifications. Due to the small size of HC, the amount of tissue from this region was insufficient for analysis. Briefly, lipids were extracted from homogenized brain samples using the Folch method. The lipid extract was then saponified to release esterified NP and IsoP. Neutral lipids were removed from the resulting mixture, using hexane, and samples were acidified to pH 3 to protonate NP and IsoP carboxylic acid groups. An internal standard, [^2^H_4_] 15-F_2t_-IsoP (Cayman Chemicals, Ann Arbor, MI, USA), was added prior to extraction with ethyl acetate. NP and IsoP were then derivatized to form pentafluorobenzyl (PFB) esters and subjected to HPLC (Agilent 1050, Santa Clara, CA, USA), using the method described by Walter et al. [33]. NP and IsoP fractions were collected, converted to trimethylsilyl ether derivatives, and quantified using GC/MS [33]. Selective ion monitoring was used for analysis at *m*/*z* 593 for NP, *m*/*z* 569 for IsoP and *m*/*z* 573 for the internal standard, [^2^H_4_] 15-F_2t_-IsoP. The inter-assay CV was 10%. 

### 2.7. Statistical Analysis

α- and γ-tocopherol and fatty acid data are expressed as mean ± standard deviation. A one-way analysis of variance (ANOVA) with Tukey’s honest significant difference (HSD) was performed to determine differences in tocopherol concentrations across brain regions. Due to significant differences in brain region tocopherol concentrations (*p* < 0.05), membrane data from each region was analyzed separately. A one-way ANOVA with Tukey’s HSD was performed for α- and γ-tocopherol as well as PUFAs (total, n-6, n-3, AA, and DHA), to determine their membrane distributions within each region. Pearson correlations were performed to determine whether region and membrane-specific α-tocopherol, membrane PUFAs, and brain region PUFA oxidation products were related, adjusting for age. We considered *r* = 0.30–0.49 to be a weak correlation, *r* = 0.50–0.69, a moderate correlation and *r* = 0.70–0.99 a strong correlation [35]. All analyses were performed using Statistical Analysis Software, SAS 9.4, with significance set at the 0.05 level.

## 3. Results

### 3.1. Tocopherol Concentrations in Rhesus Monkey Stock Diet and Serum

Tocopherol concentrations in the monkey diet and serum are presented in Table 1. In the stock diet, γ-tocopherol concentrations were ~7.5 times greater than the α-tocopherol content. The average amount of α-tocopherol consumed from the stock diet was ~2 mg/day or 0.70 µmol/kg body weight, while average γ-tocopherol intakes were ~16 mg/day or 5.3 µmol/kg body weight. Conversely, concentrations of α-tocopherol in serum were ~8 times greater than γ-tocopherol concentrations.

### 3.2. Distribution of α-Tocopherol in Brain Regions and Membranes of Adult Rhesus Monkeys

**Figure 1:**
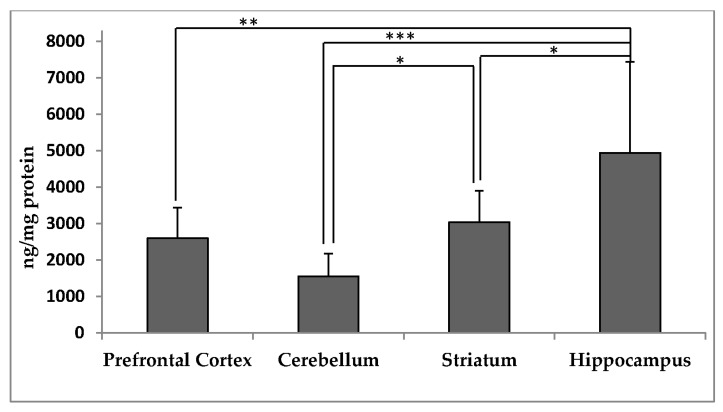
α-Tocopherol concentrations (ng/mg protein, mean ± SD) in different regions of the brain from adult rhesus macaques (*n* = 9). Asterisks indicate significant differences between brain regions according to one-way analysis of variance (ANOVA) followed by Tukey’s honest significance difference (HSD) test; * *p* < 0.05, ** *p* < 0.01, *** *p* < 0.001.

The α-tocopherol contents in the PFC, CER, ST, and HC of rhesus monkeys are presented in Figure 1. Concentrations of α-tocopherol were significantly lower in the PFC, CER and ST than in HC (*p* = 0.02), and also significantly lower in the CER than in the ST (*p* < 0.05). α-Tocopherol concentrations did not significantly differ between the ST and PFC. Concentrations of γ-tocopherol in the HC were significantly greater than in all other regions (*p* < 0.05, Figure S1). As in serum, α-tocopherol concentrations were 7–10 times greater than γ-tocopherol concentrations in the brain regions (Figure S2).

**Figure 2:**
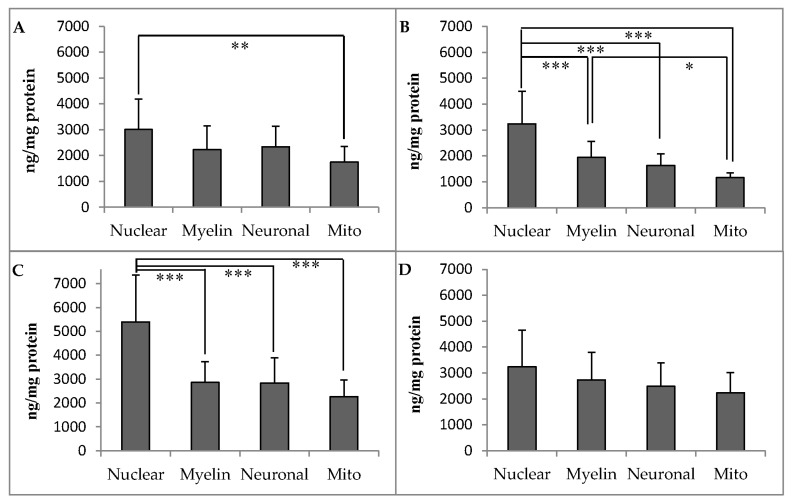
Membrane α-tocopherol concentrations (ng/mg protein, mean ± SD) in the (**A**) prefrontal cortex; (**B**) cerebellum; (**C**) striatum; and (**D**) hippocampus of adult rhesus macaques (*n* = 9). Asterisks indicate significant differences between brain regions according to one-way analysis of variance (ANOVA) followed by Tukey’s honest significant difference (HSD) test; * *p* < 0.05, ** *p* < 0.01, *** *p* < 0.001. Nuclear: nuclear membrane; myelin: myelin membranes; Neuronal: neuronal plasma membrane; Mito: mitochondrial membranes.

Membrane concentrations of α-tocopherol in the PFC, CER, ST, and HC of rhesus monkeys are presented in Figure 2A–D. The distribution of α-tocopherol among membrane types differed in a region-specific manner. In the PFC, α-tocopherol concentrations were significantly greater in nuclear membranes than in mitochondrial membranes (*p* < 0.05). In the CER, α-tocopherol concentrations were significantly greater in nuclear membranes than in all other membranes, and significantly greater in myelin than in mitochondrial membranes (*p* < 0.05). In the ST, α-tocopherol was again significantly greater in nuclear membranes than in all other membrane types (*p* < 0.05). In the HC, however, there were no significant differences in α-tocopherol concentrations across membrane types. Membrane concentrations of γ-tocopherol in brain regions were considerably lower than α-tocopherol (Figure S3A–D), and there were no significant differences among membrane types in the PFC and HC. However, in the CER and ST, γ-tocopherol concentrations were significantly greater in nuclear membranes than in all other membrane types (*p* ≤ 0.02).

### 3.3. Distribution of Membrane PUFAs in Different Brain Regions of Adult Rhesus Macaques

Membrane concentrations of total PUFA, n-6, n-3, AA, and DHA in the PFC, CER, ST, and HC of rhesus monkeys are presented in Table 2. In the PFC and ST, AA and total n-6 PUFA concentrations were highest in nuclear membranes, intermediate in myelin and neuronal membranes, and lowest in mitochondrial membranes (*p* < 0.05). However, in the CER, AA was similar among all membranes, except mitochondrial membranes, where concentrations were lower than in all other membrane fractions. In the HC, mean values were highest in myelin and nuclear membranes, followed by neuronal and mitochondrial membranes (*p* < 0.05).

In the PFC, DHA, n-3 PUFAs, and the total PUFA concentration were lowest in mitochondrial membranes (*p* < 0.05), but did not differ in the other membranes. In the CER, DHA and n-3 PUFAs were highest in neuronal membranes and myelin, followed by nuclear membranes, and lowest in mitochondrial membranes (*p* < 0.05). In this region, the total PUFA content was lowest in mitochondrial membranes compared to the other membranes (*p* < 0.05). In both the ST and HC, PUFA n-3 and DHA were highest in myelin membranes, intermediate in neuronal and nuclear membranes, and lowest in mitochondrial membranes (*p* < 0.05). In the ST, total PUFA concentrations differed across all membranes (nuclear > myelin > neuronal > mitochondrial, *p* < 0.05). In the HC, total PUFAs were highest in myelin, intermediate in neuronal and nuclear membranes, and lowest in mitochondrial membranes (*p* < 0.05).

While the absolute concentration of fatty acids provides a measurement of the amount of each fatty acid, independent of other fatty acids, mole % data measures the relative importance of a fatty acid against the total fatty acid concentration. Thus, the membrane distribution of fatty acids differs, depending on whether fatty acids are expressed as mole % or µg/mg protein. Membrane fatty acid profiles among brain regions, expressed as mole %, are reported in the supplementary material (Tables S1–S4). Briefly, in the PFC, CER and ST, the mole % AA was highest in mitochondrial membranes, intermediate in neuronal and myelin membranes, and lowest in nuclear membranes (Tables S1–S3, *p* < 0.05). In the PFC, DHA was distributed similarly to AA among membranes (Table S1, *p* < 0.05). In the CER, the mole % DHA was highest in mitochondrial membranes, followed by neuronal membranes, followed by myelin membranes, and lowest in nuclear membranes (Table S2, *p* < 0.05). In the ST, the mole % DHA was highest in mitochondrial and myelin membranes, intermediate in neuronal membranes, and lowest in nuclear membranes (Table S3, *p* < 0.05). In the HC, the mole % AA was highest in mitochondrial membranes, but did not differ among the other membranes (Table S4, *p* < 0.05). The mole % DHA in this region was lowest in nuclear membranes but did not differ among other membrane types (*p* < 0.05). 

### 3.4. Relationship between Membrane α-Tocopherol and PUFA Concentrations in Brain Regions

The association between the membrane-specific α-tocopherol and PUFA concentrations in the PFC, CER, ST, and HC is presented in Table 3. In all regions, except the HC, α-tocopherol concentrations were positively associated with all PUFA concentrations—including AA, DHA, n-6 and n-3 PUFA and total PUFA—in nuclear membranes (*p* < 0.05), with the single exception of DHA in the CER. No other significant associations were observed in the CER. In the PFC, α-tocopherol concentrations were also associated with all PUFAs in neuronal plasma membranes (*p* < 0.05). In the ST, α-tocopherol was significantly associated with all PUFAs in all membrane types (*p* < 0.05). In the HC, α-tocopherol was associated with all PUFAs in myelin, but only DHA and total PUFA n–3 in mitochondrial membranes (*p* < 0.05). No significant associations were observed in nuclear and neuronal plasma membranes from this region.

### 3.5. Relationship between Membrane α-Tocopherol and PUFA Oxidation Products in Brain Regions

**Figure 3:**
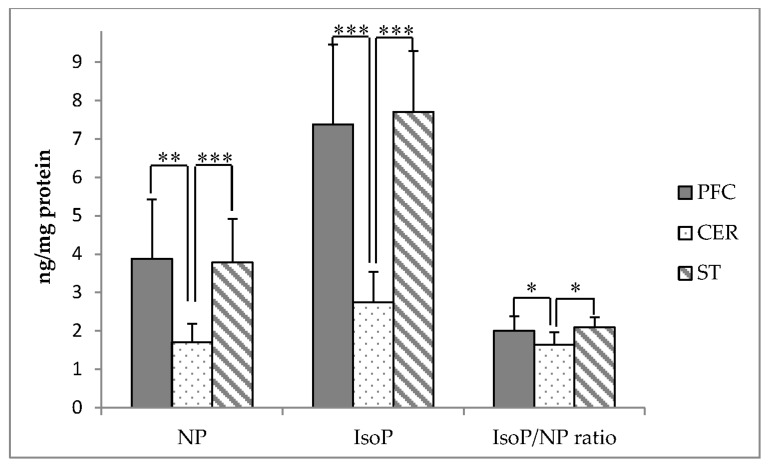
Neuroprostane (NP) and isoprostane (IsoP) concentrations (ng/mg protein) in the prefrontal cortex (PFC), cerebellum (CER), and striatum (ST) of adult rhesus macaques (*n* = 9). Asterisks indicate significant differences between brain regions according to one-way ANOVA followed by Tukey’s HSD test; * *p* < 0.05, ** *p* < 0.01, *** *p* < 0.001.

Concentrations of DHA oxidation products (NP) and AA oxidation products (IsoP) among the PFC, CER, and ST are presented in Figure 3. NP and IsoP were significantly lower in the CER compared to both the PFC and ST (*p* < 0.01). The ratio of IsoP/NP was also significantly lower in the CER, compared to the other two regions (*p* < 0.05). NP, IsoP, and the ratio of IsoP/NP did not differ between the PFC and ST.

The cross-sectional relationship between region and membrane-specific α-tocopherol concentrations and brain PUFA oxidation products in the PFC, CER, and ST is presented in Table 4. NP concentrations (pg/µg DHA), measured in whole tissue, were not significantly associated with α-tocopherol concentrations (ng/µg DHA) in any membrane type or brain region. Similarly, IsoP concentrations (pg/µg AA) were not associated with α-tocopherol (ng/µg AA) in any individual membrane type. However, AA oxidation products were inversely associated with concentrations of α-tocopherol in whole tissue, in both the PFC and ST (*p* < 0.05). PUFA oxidation products were not associated with γ-tocopherol concentrations in any membrane or region (data not shown).

## 4. Discussion

This study is the first to report the distribution of α-tocopherol and γ-tocopherol in multiple subcellular membrane types (nuclear, myelin, neuronal, and mitochondrial) for multiple brain regions in the primate brain. We also directly investigated the relationship between membrane α-tocopherol concentration and both membrane PUFA content and brain PUFA oxidation. We report that α-tocopherol was differentially distributed among the membranes, with the highest concentrations found in nuclear membranes for all regions tested, except the HC. In the PFC and ST, AA and total n-6 PUFA distribution among membrane types were similar to that of α-tocopherol, and total α-tocopherol concentrations in these regions were inversely related to AA oxidation products. 

### 4.1. α-Tocopherol Distribution in Brain Regions of Adult Rhesus Monkeys

Although no other studies have reported on the concentration of α-tocopherol in monkey brain regions, our results are similar to α-tocopherol concentrations reported in different regions of the adult human brain [6,36], but slightly greater than those reported for human infants [37] and rats [20]. In the current study, the CER had the lowest α-tocopherol concentrations among the brain regions analyzed. This is consistent with previous findings in brain tissue from human adults and centenarians [6,36] as well as rats [20]. In the rat brain, researchers have also observed that the uptake of α-tocopherol is greatest in the CER, despite this region having the lowest steady-state amounts of α-tocopherol, suggesting a more rapid turnover of α-tocopherol in the CER, compared to other regions [20]. Alternatively, this observation in primates may be due to differences in α-tocopherol transfer protein (α-TTP) and/or tocopherol-associated protein (TAP) expression among brain regions [38,39]. In human infants, there were no differences in α-tocopherol concentrations across brain regions tested [37], suggesting that α-tocopherol distribution in the brain might differ across the lifespan. However, that study did not analyze the CER. Future studies investigating the mechanisms underlying the differential distribution of α-tocopherol across the brain tissue of adult primates are warranted.

Our detection of γ-tocopherol at significantly lower concentrations than α-tocopherol is also consistent with findings from studies in human brain tissue [36,37], but differs from rodent studies, in which only α-tocopherol has been detected [20,40]. The distribution pattern of γ-tocopherol among brain regions and membrane fractions was similar to that of α-tocopherol, indicating that tocopherol delivery to brain regions may depend on both serum levels and brain region-specific factors. 

### 4.2. Accumulation of α-Tocopherol in Brain Membranes of Adult Rhesus Monkeys

The distribution of α-tocopherol among membrane types differed in a region-specific manner. However, α-tocopherol concentrations were generally greater in nuclear membranes, compared to other membranes, in each region, except for the HC. The enrichment of α-tocopherol in nuclear membranes suggests that α-tocopherol may play a role in nuclear-associated functions in the brain. This role may be particularly important in the ST and CER, where preferential accumulation of α-tocopherol in the nuclear membrane was most apparent. Accumulating evidence from a number of in vitro and ex vivo studies indicates that α-tocopherol can modulate the expression of cell signaling, cell cycle regulation, and pro-inflammatory genes [15,16,17,18,41]. In mice and rats, dietary and supplemental α-tocopherol or the combination of α- and γ-tocopherol has been shown to modulate the expression of genes involved in apoptosis, lipid biosynthesis, adenosine triphosphate (ATP) biosynthesis, and immune/inflammatory responses in the aging brain [42,43]. Additionally, the expression of genes encoding synaptic proteins, protein kinase C family members, and myelin proteins were decreased in transgenic mice deficient in α-TTP [44]. These results may have important implications for the potential role of α-tocopherol in protecting against cognitive impairment and dementia, given that microarray studies have demonstrated that dysregulation of genes involved in inflammation, synaptic signaling, and neuronal apoptosis alter brain cell function and contribute to the pathogenesis of these conditions [45,46,47]. Future studies investigating the effect of α-tocopherol enrichment in nuclear membranes and changes in gene expression in the primate brain are needed to gain a better understanding of why α-tocopherol may accumulate in this membrane. 

Membrane α-tocopherol and PUFA concentrations were found to be positively associated with one another, particularly in the PFC and ST, and to a lesser extent in the CER and HC. This finding is consistent with previous studies, which demonstrated that α-tocopherol accumulates in PUFA-rich membrane domains [48,49]. No significant relationship was observed between α-tocopherol and brain PUFA oxidation products in individual membrane fractions. However, concentrations of both DHA and AA oxidation products (as measured by NP and IsoP, respectively), as well as the relative amount of AA oxidation products to DHA oxidation products (IsoP/NP ratio) were lower in the CER compared to other brain regions, similar to α-tocopherol. Additionally, α-tocopherol concentrations in whole tissue were inversely associated with AA oxidation products (IsoP) in both the PFC and ST. These are the only two brain regions where concentrations of both α-tocopherol and AA were highest in nuclear membranes, intermediate in myelin and neuronal membranes, and lowest in mitochondrial membranes. In contrast, α-tocopherol concentrations were not correlated with DHA oxidation products, as measured by NP levels. These results suggest that (1) α-tocopherol may accumulate in regions with higher PUFA oxidation, particularly AA oxidation relative to DHA oxidation; and (2) α-tocopherol concentrations within these regions of relative higher accumulation may be associated with lower AA oxidation. This is consistent with previous rodent studies, which demonstrated that supplementation with α-tocopherol is more effective at decreasing AA oxidation than DHA oxidation [50,51]. Taken together, our findings indicate that, although α-tocopherol accumulates in membranes, rich in both n-6 and n-3 PUFAs, it may be associated with preferentially protecting AA from oxidative damage. Our observation that only concentrations of α-tocopherol in whole tissue were associated with AA oxidation suggests that the free-radical scavenging function of this nutrient may not be specific to a particular membrane type. Therefore, the contribution of total α-tocopherol concentrations within regions may have the strongest relationship to total AA oxidation in brain regions compared to individual membranes. However, only whole tissue concentrations of AA and DHA oxidation products were determined in these studies as it is currently not feasible to measure membrane-specific concentrations, due to limitations in methodology. Therefore, the relationship between α-tocopherol and PUFA oxidation products within each membrane type remains unknown. Our α-tocopherol result differs from our previous findings that membrane-specific concentrations of the antioxidant, lutein, are associated with total DHA oxidation products, but not total AA oxidation products in rhesus monkey brains [23]. One limitation of both studies is that neither accounts for the potential synergistic effects of other antioxidants present in the brain and their influence on DHA and AA oxidation. Therefore, future studies measuring a more comprehensive profile of antioxidants in the brain and their associations with PUFA oxidation, are needed. Another limitation of this study is the small sample size. Our results need to be replicated in a larger sample population. However, our study provides an important first step in characterizing previously unknown relationships between membrane-associated antioxidants and PUFA oxidation in the primate brain and can guide future investigations into the antioxidant functions of α- and γ-tocopherol in the brain.

Our findings support an antioxidant-associated function of α-tocopherol towards AA, but we cannot rule out the possibility that non-antioxidant functions underlie the association between these two nutrients. Previous studies have demonstrated that α-tocopherol can significantly inhibit the activity of phospholipase A2 [41,52], which is primarily responsible for cleavage and release of AA from membrane phospholipids. Therefore, it is possible that the similarity in membrane distribution between α-tocopherol and AA may reflect not only a role of α-tocopherol in inhibiting AA oxidation, but also a role in the modulation of AA cleavage and release from membranes. Future studies, investigating the relationship between membrane α-tocopherol levels and phospholipase A2 activity in regions of the primate brain, are needed, to better understand the contribution of this potential mechanism to the overall function of α-tocopherol in the primate brain. 

## 5. Conclusions

In conclusion, we found that dietary α-tocopherol was higher in the HC than in the PFC, CER or ST in the non-human primate brain. Within membrane types, α-tocopherol showed preferential accumulation in nuclear membranes compared to other membrane types, except in the HC. We speculate that this observation may be indicative of a role of α-tocopherol in nuclear functions in brain cells. Additionally, we observed that α-tocopherol is positively related to PUFA concentrations in membranes, but only concentrations of AA were distributed similarly to α-tocopherol among membrane types. Finally, whole tissue, but not membrane, α-tocopherol concentrations were significantly associated with AA oxidation products, in both the PFC and ST. Thus, correlations between α-tocopherol and PUFA concentrations may be associated with a protective role of α-tocopherol against AA oxidation, but may also reflect a role of α-tocopherol in inhibiting AA release from membrane phospholipids. Collectively, our study provides insight into the accumulation of dietary α-tocopherol in the primate brain, which may have important implications regarding its functions in this tissue.

## Figures and Tables

**Table 1:** Tocopherol Concentrations (mean ± SD) in Monkey Stock Diet * and Serum (*n* = 7).

	α-Tocopherol	γ-Tocopherol
Stock Diet (µmol/kg)	23 ± 1.2	171 ± 34
Serum (µmol/L)	20.8 ± 8.3	2.5 ± 0.6

* Sample run in triplicate.

**Table 2:** Membrane polyunsaturated fatty acid concentration (µg/mg protein, mean ± SD) in the prefrontal cortex, cerebellum, striatum, and hippocampus of adult rhesus macaques (*n* = 9).

Region		Nuclear	Myelin	Neuronal	Mitochondrial
**Prefrontal Cortex**	Arachidonic Acid	123.5 ± 30.0 ^a^	99.3 ± 30.5 ^b^	96.44 ± 20.0 ^b^	28.0 ± 16.3 ^c^
	PUFA n-6	291.7 ± 77.5 ^a^	204.9 ± 48.9 ^b^	201.6 ± 63.5 ^b^	49.2 ± 27.6 ^c^
	Docosahexaenoic acid	151.9 ± 37.2 ^a^	180.6 ± 48.9 ^a^	175.8 ± 54.8 ^a^	35.3 ± 19.2 ^b^
	PUFA n-3	164.2 ± 44.6 ^a^	187.6 ± 51.7 ^a^	182.9 ± 56.8 ^a^	36.2 ± 19.8 ^b^
	Total PUFA	455.9 ± 120.6 ^a^	392.4 ± 99.9 ^a^	384.5 ± 120.1 ^a^	85.4 ± 47.1 ^b^
**Cerebellum**	Arachidonic Acid	91.4 ± 19.1 ^a^	84.4 ± 32.8 ^a^	95.9 ± 20.4 ^a^	15.1 ± 5.8 ^b^
	PUFAn-6	213.0 ± 40.2 ^a^	166.1 ± 59.3 ^b^	187.6 ± 38.0 ^a,b^	30.3 ± 11.4 ^c^
	Docosahexaenoic acid	136.0 ± 26.5 ^a^	178.2 ± 59.9 ^b^	199.8 ± 39.7 ^b^	27.8 ± 10.2 ^c^
	PUFA n-3	148.8 ± 30.0 ^a^	185.9 ± 63.3 ^b^	207.3 ± 40.9 ^b^	28.7 ± 10.5 ^c^
	Total PUFA	361.8 ± 69.4 ^a^	352.0 ± 121.4 ^a^	394.9 ± 76.7 ^a^	59.0 ± 21.9 ^b^
**Striatum**	Arachidonic Acid	221.5 ± 65.4 ^a^	156.2 ± 20.5 ^b^	143.9 ± 26.5 ^b^	30.5 ± 13.3 ^c^
	PUFA n-6	525.2 ± 177.7 ^a^	315.5 ± 46.4 ^b^	289.0 ± 47.6 ^b^	52.9 ± 22.1 ^c^
	Docosahexaenoic acid	196.9 ± 36.1 ^a^	257.7 ± 35.0 ^b^	201.0 ± 37.2 ^a^	37.3 ± 17.4 ^c^
	PUFA n-3	224.0 ± 43.9 ^a^	268.7 ± 36.7 ^b^	211.7 ± 38.5 ^a^	38.4 ± 17.8 ^c^
	Total PUFA	749.2 ± 217.5 ^a^	584.2 ± 81.6 ^b^	500.7 ± 85.1 ^c^	91.4 ± 39.6 ^d^
**Hippocampus**	Arachidonic Acid	208.0 ± 54.6 ^a,b^	247.1 ± 119.7 ^a^	170.6 ± 50.4 ^b^	21.6 ± 6.6 ^c^
	PUFA n-6	452.0 ± 126.0 ^a^	519.7 ± 226.7 ^a^	340.0 ± 104.9 ^b^	37.0 ± 10.9 ^c^
	Docosahexaenoic acid	144.2 ± 40.9 ^a^	325.2 ± 118.7 ^b^	201.5 ± 63.4 ^c^	16.7 ± 5.5 ^d^
	PUFA n-3	191.6 ± 54.2 ^a^	347.7 ± 127.5 ^b^	219.4 ± 69.7 ^a^	18.2 ± 5.5 ^c^
	Total PUFA	643.6 ± 177.5 ^a,b^	867.5 ± 351.2 ^a^	559.5 ± 173.8 ^b^	55.2 ± 16.1 ^c^

Means with different superscripts (a, b, c, d) in each row are significantly different, according to one-way analysis of variance (ANOVA) followed by Tukey’s honest significance difference (HSD) test (*p* < 0.05). PUFA: polyunsaturated fatty acids.

**Table 3:** Partial correlations between membrane α-tocopherol (ng/mg protein) and PUFA concentrations (µg/mg protein) from different brain regions of rhesus macaques (*n* = 9).

Region		Nuclear	Myelin	Neuronal	Mitochondrial
Prefrontal Cortex	Arachidonic acid	0.68 **	0.69 **	0.78 **	--
	PUFA n-6	0.70 **	0.61	0.82 **	--
	Docosahexaenoic acid	0.70 **	0.55	0.94 ***	--
	PUFA n-3	0.71 **	0.54	0.94 ***	--
	Total PUFA	0.71 **	0.58	0.91 ***	--
Cerebellum	Arachidonic acid	0.78 **	0.43	0.52	0.53
	PUFA n-6	0.85 ***	0.47	0.58	0.57
	Docosahexaenoic acid	0.61	0.40	0.63	0.56
	PUFA n-3	0.70 **	0.41	0.62	0.56
	Total PUFA	0.76 **	0.44	0.60	0.57
Striatum	Arachidonic acid	0.89 ***	0.81 **	0.86 ***	0.80 **
	PUFA n-6	0.81 **	0.76 **	0.84 ***	0.75 **
	Docosahexaenoic acid	0.89 ***	0.81 **	0.80 **	0.80 **
	PUFA n-3	0.91 ***	0.80 **	0.79 **	0.79 **
	Total PUFA	0.85 ***	0.79 **	0.83 **	0.77 **
Hippocampus	Arachidonic acid	0.60	0.71 **	0.48	0.61
	PUFA n-6	0.64	0.79 **	0.50	0.58
	Docosahexaenoic acid	0.61	0.70 **	0.57	0.79 **
	PUFA n-3	0.67	0.70 **	0.56	0.77 **
	Total PUFA	0.66	0.76 **	0.53	0.66

Values are partial correlation coefficients (*r* values) adjusted for age; ** *p* < 0.05, *** *p* < 0.01. --: *r* values below 0.40 are not shown.

**Table 4:** Partial correlations between membrane α-tocopherol and neuroprostanes and isoprostanes from different brain regions of rhesus macaques (*n* = 9).

	Total	Nuclear	Myelin	Neuronal	Mitochondrial
*Neuroprostanes*					
Prefrontal Cortex	−0.66 *	--	--	--	--
Cerebellum	--	--	--	--	--
Striatum	−0.43	−0.62 *	--	--	--
*Isoprostanes*					
Prefrontal Cortex	−0.74 **	−0.47	--	--	--
Cerebellum	--	−0.66 *	--	--	−0.61 *
Striatum	−0.73 **	--	−0.64 *	-0.50	--

Values are partial correlation coefficients (*r* values) adjusted for age; ** *p* < 0.05, * *p* < 0.1. --: *r* values below 0.40 are not shown.

## Supplementary Materials

The following are available online at www.mdpi.com/2076-3921/6/4/97/s1, Figure S1. Mean γ-tocopherol concentrations (ng/mg protein, ±SD) in different regions of the brain from adult rhesus macaques (*n* = 9), Figure S2. Mean (±SD) ratio of α-tocopherol/γ-tocopherol in different regions of the brain from adult rhesus macaques (*n* = 9). Figure S3. Membrane γ-tocopherol concentrations (ng/mg protein, mean ± SD) in (A) prefrontal cortex (B) cerebellum (C) striatum (D) hippocampus of adult rhesus macaques (*n* = 9), Table S1. Mean (±SD) mole percent fatty acids in prefrontal cortex membranes of adult rhesus macaques (*n* = 9), Table S2. Mean (±SD) mole percent fatty acids in cerebellar membranes of adult rhesus macaques (*n* = 9), Table S3. Mean (±SD) mole percent fatty acids in striatal membranes of adult rhesus macaques (*n* = 9), Table S4. Mean (±SD) mole percent fatty acids in hippocampal membranes of adult rhesus macaques (*n* = 9).
