# Supplementary material for: The Subcellular Distribution of Alpha-Tocopherol in the Adult Primate Brain and Its Relationship with Membrane Arachidonic Acid and Its Oxidation Products
Source: Antioxidants (Basel). 2017 Nov 26;6(4):97. doi: 10.3390/antiox6040097 (PMC5745507; doi:10.3390/antiox6040097)
Supplement: Supplementary file 1 [file antioxidants-06-00097-s001.zip › Supplementary material/Supplementary figures.docx]

**Figure S1**. Mean (±SD) γ-tocopherol concentrations (ng/mg protein, ± SD) in different regions of the brain from adult rhesus macaque (n=9).

***

***

***

Asterisks indicate significant differences between brain regions according to one-way ANOVA followed by Tukey’s HSD test; *P<0.05, **P<0.01, ***P<0.001

**Figure S2**. Mean (±SD) ratio of α-tocopherol/γ-tocopherol in different regions of the brain from adult rhesus macaque (n=9).

*

Asterisks indicate significant differences between brain regions according to one-way ANOVA followed by Tukey’s HSD test; *P<0.05, **P<0.01, ***P<0.001

**Figure S3**. Membrane γ-tocopherol concentrations (ng/mg protein, mean ± SD) in (A) prefrontal cortex (B) cerebellum (C) striatum (D) hippocampus of adult rhesus macaque (n=9).

**A**

**

**

*


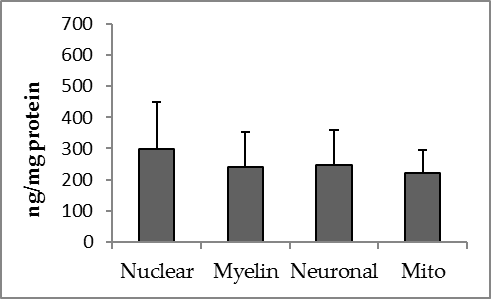

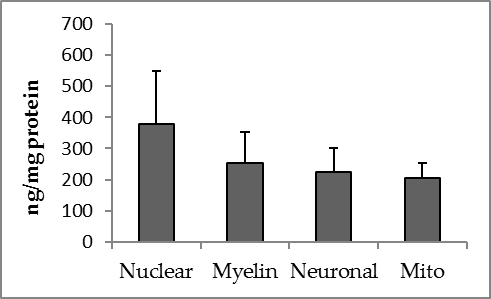

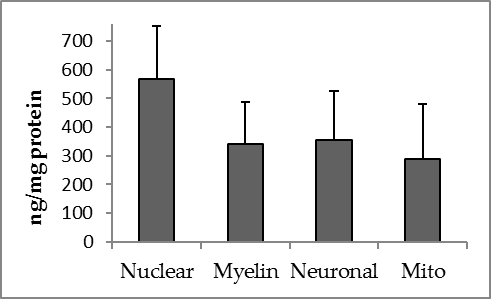

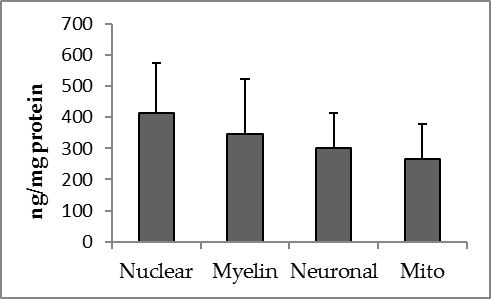


**B**

**

**C**

**D**

**

*

Asterisks indicate significant differences between brain regions according to one-way ANOVA followed by Tukey’s HSD test; *P<0.05, **P<0.01, ***P<0.001Neuronal: neuronal plasma membrane; Mito: mitochondrial membranes
