# Supplementary material for: The Subcellular Distribution of Alpha-Tocopherol in the Adult Primate Brain and Its Relationship with Membrane Arachidonic Acid and Its Oxidation Products
Source: Antioxidants (Basel). 2017 Nov 26;6(4):97. doi: 10.3390/antiox6040097 (PMC5745507; doi:10.3390/antiox6040097)
Supplement: Supplementary file 1 [file antioxidants-06-00097-s001.zip › Supplementary material/Supplementary tables.docx]

**Table S1**. Mean (±SD) mole percent fatty acids in prefrontal cortex membranes of adult rhesus macaques (n=9)

|  | **Nuclear** | **Myelin** | **Neuronal** | **Mitochondrial** |
| --- | --- | --- | --- | --- |
| *Total SFA* | 48.53 ± 1.52^a^ | 51.14 ± 3.21^b^ | 51.28 ± 1.01^b^ | 43.98 ± 3.28^c^ |
| *Total MUFA* | 27.61 ± 1.70^a^ | 20.10 ± 1.37^b^ | 20.28 ± 0.57^b^ | 21.79 ± 0.98^c^ |
| *Total PUFAn-6* | 15.25 ± 0.34^a^ | 15.30 ± 1.84^a,b^ | 15.07 ± 0.57^b^ | 20.13 ± 2.25^c^ |
| 18:2n-6 (LA) | 0.78 ± 0.09^a^ | 1.04 ± 0.70^b^ | 0.95 ± 0.09^b^ | 2.71 ± 0.75^c^ |
| 18:3n-6 (GLA) | 0.20 ± 0.28^a^ | 0.38 ± 0.35^a^ | 0.32 ± 0.42^a^ | 0.47 ± 0.50^a^ |
| 20:2n-6 | 0.16 ± 0.03^a^ | 0.09 ± 0.03^b^ | 0.09 ± 0.04^b^ | 0.12 ± 0.01^c^ |
| 20:3n-6 (DGLA) | 1.49 ± 0.24^a^ | 1.08 ± 0.31^b^ | 1.03 ± 0.15^b^ | 1.31 ± 0.53^a^ |
| 20:4n-6 (AA) | 6.69 ± 0.35^a^ | 7.72 ± 1.50^b^ | 7.40 ± 0.28^b^ | 11.62 ± 1.69^c^ |
| 22:2n-6 | 0.09 ± 0.05^a^ | 0.03 ± 0.01^b^ | 0.03 ± 0.01^b^ | 0.02 ± 0.01^b^ |
| 22:4n-6 | 5.21 ± 0.40^a^ | 3.88 ± 0.47^b^ | 4.15 ± 0.19^c^ | 2.86 ± 0.51^d^ |
| 22:5n-6 | 0.64 ± 0.23^a^ | 1.08 ± 0.16^b^ | 1.10 ± 0.15^b^ | 1.03 ± 0.12^b^ |
| *Total PUFAn-3* | 8.27 ± 0.75^a^ | 13.06 ± 0.59^b^ | 13.07 ± 0.42^b^ | 13.74 ± 0.56^c^ |
| 18:3n-3 (ALA) | 0.06 ± 0.12^a^ | 0.06 ± 0.09^a^ | 0.08 ± 0.11^a^ | 0.04 ± 0.03^a^ |
| 18:4n-3 (SDA) | 0.25 ± 0.24^a^ | 0.12 ± 0.06^a^ | 0.16 ± 0.09^a^ | 0.08 ± 0.08^a^ |
| 20:5n-3 (EPA) | 0.02 ± 0.02^a^ | 0.02 ± 0.01^a^ | 0.03 ± 0.01^a^ | 0.03 ± 0.01^a^ |
| 22:5n-3 (DPA) | 0.30 ± 0.14^a^ | 0.29 ± 0.07^a^ | 0.28 ± 0.07^a^ | 0.20 ± 0.05^b^ |
| 22:6n-3 (DHA) | 7.63 ± 0.55^a^ | 12.57 ± 0.57^b^ | 12.53 ± 0.49^b^ | 13.39 ± 0.51^c^ |
| *Total Trans* | 0.34 ± 0.12^a^ | 0.40 ± 0.14^a^ | 0.31 ± 0.13^a^ | 0.35 ± 0.05^a^ |

Means with different superscripts across membrane types are significantly different according to Tukey’s HSD test (P<0.05).

SFA: saturated fatty acids, MUFA: monounsaturated fatty acids, PUFAn6: n-6 polyunsaturated fatty acids, LA: linoleic acid, GLA: gamma-linolenic acid, DGLA: dihomo-gamma-linolenic acid, AA: arachidonic acid, PUFAn3: n-3 polyunsaturated fatty acids, ALA: α-linolenic acid, SDA: stearidonic acid, EPA: eicosapentaenoic acid, DPA: docosapentaenoic acid, DHA: docosahexaenoic acid, *trans*: *trans* fatty acids

**Table S2**. Mean (±SD) mole percent fatty acids in cerebellar membranes of adult rhesus macaques (n=9)

|  | **Nuclear** | **Myelin** | **Neuronal** | **Mitochondrial** |
| --- | --- | --- | --- | --- |
| *Total SFA* | 49.01 ± 2.02^a^ | 51.68 ± 0.38^b^ | 50.94 ± 0.95^c^ | 43.57 ± 1.43^d^ |
| *Total MUFA* | 29.02 ± 1.26^a^ | 22.66 ± 1.01^b,c^ | 21.83 ± 1.34^b^ | 23.13 ± 0.79^c^ |
| *Total PUFAn-6* | 12.93 ± 1.01^a^ | 12.37 ± 0.96^b^ | 13.22 ± 0.63^a^ | 17.53 ± 0.85^c^ |
| 18:2n-6 (LA) | 0.96 ± 0.13^a^ | 1.05 ± 0.13^a^ | 1.27 ± 0.11^b^ | 3.16 ± 0.28^c^ |
| 18:3n-6 (GLA) | 0.38 ± 0.29^a^ | 0.56 ± 0.24^a^ | 0.48 ± 0.29^a^ | 0.65 ± 0.42^a^ |
| 20:2n-6 | 0.31 ± 0.24^a^ | 0.17 ± 0.02^b^ | 0.17 ± 0.02^b^ | 0.22 ± 0.03^a^ |
| 20:3n-6 (DGLA) | 2.06 ± 0.28^a^ | 1.50 ± 0.20^b^ | 1.57 ± 0.22^b^ | 2.41 ± 0.36^a^ |
| 20:4n-6 (AA) | 5.65 ± 0.58^a^ | 6.35 ± 0.97^b^ | 6.82 ± 0.51^b^ | 8.71 ± 0.55^c^ |
| 22:2n-6 | 0.14 ± 0.05^a^ | 0.04 ± 0.02^b^ | 0.04 ± 0.01^b^ | 0.05 ± 0.02^b^ |
| 22:4n-6 | 3.07 ± 0.44^a^ | 2.22 ± 0.17^b^ | 2.34 ± 0.17^b^ | 1.79 ± 0.13^c^ |
| 22:5n-6 | 0.36 ± 0.07^a^ | 0.47 ± 0.09^b^ | 0.52 ± 0.12^b^ | 0.53 ± 0.09^b^ |
| *Total PUFAn-3* | 8.56 ± 0.65^a^ | 13.03 ± 0.40^b^ | 13.76 ± 1.01^c^ | 15.38 ± 0.63^d^ |
| 18:3n-3 (ALA) | 0.01 ± 0.01^a^ | 0.08 ± 0.13^b^ | 0.02 ± 0.01^a^ | 0.09 ± 0.11^b^ |
| 18:4n-3 (SDA) | 0.30 ± 0.13^a^ | 0.08 ± 0.07^b^ | 0.11 ± 0.06^b^ | 0.05 ± 0.04^b^ |
| 20:5n-3 (EPA) | 0.05 ± 0.03^a^ | 0.05 ± 0.01^a^ | 0.05 ± 0.01^a^ | 0.07 ± 0.02^b^ |
| 22:5n-3 (DPA) | 0.41 ± 0.08^a^ | 0.33 ± 0.03^b^ | 0.34 ± 0.04^b^ | 0.28 ± 0.04^c^ |
| 22:6n-3 (DHA) | 7.78 ± 0.60^a^ | 12.49 ± 0.40^b^ | 13.24 ± 1.07^c^ | 14.88 ± 0.62^d^ |
| *Total Trans* | 0.48 ± 0.68^a,b^ | 0.27 ± 0.04^b^ | 0.25 ± 0.05^b^ | 0.39 ± 0.08^a^ |

Means with different superscripts across membrane types are significantly different according to Tukey’s HSD test (P<0.05).

SFA: saturated fatty acids, MUFA: monounsaturated fatty acids, PUFAn6: n-6 polyunsaturated fatty acids, LA: linoleic acid, GLA: gamma-linolenic acid, DGLA: dihomo-gamma-linolenic acid, AA: arachidonic acid, PUFAn3: n-3 polyunsaturated fatty acids, ALA: α-linolenic acid, SDA: stearidonic acid, EPA: eicosapentaenoic acid, DPA: docosapentaenoic acid, DHA: docosahexaenoic acid, *trans*: *trans* fatty acids

**Table S3**. Mean (±SD) mole percent fatty acids in striatal membranes of adult rhesus macaques (n=9)

|  | **Nuclear** | **Myelin** | **Neuronal** | **Mitochondrial** |
| --- | --- | --- | --- | --- |
| *Total SFA* | 48.11 ± 2.45^a^ | 52.30 ± 0.73^b^ | 49.69 ± 0.90^a^ | 45.93 ± 7.10^c^ |
| *Total MUFA* | 29.40 ± 2.66^a^ | 19.89 ± 1.08^b^ | 22.24 ± 1.47^c^ | 21.41 ± 2.35^c^ |
| *Total PUFAn-6* | 15.42 ± 1.00^a^ | 15.19 ± 0.48^a^ | 16.37 ± 0.42^b^ | 19.10 ± 3.23^b^ |
| 18:2n-6 (LA) | 0.62 ± 0.12^a^ | 0.55 ± 0.08^a^ | 0.77 ± 0.12^b^ | 2.06 ± 0.41^c^ |
| 18:3n-6 (GLA) | 0.25 ± 0.33^a^ | 0.22 ± 0.32^a^ | 0.16 ± 0.29^a^ | 0.16 ± 0.41^a^ |
| 20:2n-6 | 0.30 ± 0.45^a^ | 0.10 ± 0.02^b^ | 0.11 ± 0.02^b,c^ | 0.30 ± 0.53^a,c^ |
| 20:3n-6 (DGLA) | 1.75 ± 0.30^a^ | 1.14 ± 0.21^b^ | 1.43 ± 0.18^b^ | 1.99 ± 0.70^a^ |
| 20:4n-6 (AA) | 6.81 ± 0.91^a^ | 7.76 ± 0.48^b^ | 8.35 ± 0.48^b^ | 11.21 ± 2.35^c^ |
| 22:2n-6 | 0.08 ± 0.03^a^ | 0.02 ± 0.01^b^ | 0.04 ± 0.02^b^ | 0.03 ± 0.02^b^ |
| 22:4n-6 | 5.06 ± 0.54^a^ | 4.46 ± 0.23^b^ | 4.67 ± 0.15^b^ | 2.63 ± 0.51^c^ |
| 22:5n-6 | 0.54 ± 0.18^a^ | 0.93 ± 0.15^b^ | 0.84 ± 0.17^b,c^ | 0.73 ± 0.17^c^ |
| *Total PUFAn-3* | 6.59 ± 1.29^a^ | 12.37 ± 0.52^b^ | 11.41 ± 0.66^c^ | 12.93 ± 2.71^b^ |
| 18:3n-3 (ALA) | 0.03 ± 0.02^a^ | 0.01 ± 0.01^b^ | 0.01 ± 0.01^b^ | 0.05 ± 0.06^a^ |
| 18:4n-3 (SDA) | 0.24 ± 0.18^a^ | 0.10 ± 0.05^a^ | 0.11 ± 0.10^a^ | 0.07 ± 0.05^a^ |
| 20:5n-3 (EPA) | 0.04 ± 0.01^a^ | 0.05 ± 0.02^b^ | 0.05 ± 0.02^b^ | 0.06 ± 0.02^b^ |
| 22:5n-3 (DPA) | 0.48 ± 0.08^a^ | 0.37 ± 0.10^b^ | 0.43 ± 0.07^a,b^ | 0.21 ± 0.05^c^ |
| 22:6n-3 (DHA) | 5.80 ± 1.33^a^ | 11.85 ± 0.49^b^ | 10.81 ± 0.68^c^ | 12.55 ± 2.75^b^ |
| *Total Trans* | 0.48 ± 0.53^a,c^ | 0.25 ± 0.05^b^ | 0.29 ± 0.06^a,b^ | 0.64 ± 0.80^c^ |

Means with different superscripts across membrane types are significantly different according to Tukey’s HSD test (P<0.05).

SFA: saturated fatty acids, MUFA: monounsaturated fatty acids, PUFAn6: n-6 polyunsaturated fatty acids, LA: linoleic acid, GLA: gamma-linolenic acid, DGLA: dihomo-gamma-linolenic acid, AA: arachidonic acid, PUFAn3: n-3 polyunsaturated fatty acids, ALA: α-linolenic acid, SDA: stearidonic acid, EPA: eicosapentaenoic acid, DPA: docosapentaenoic acid, DHA: docosahexaenoic acid, *trans*: *trans* fatty acids

**Table S4**. Mean (±SD) mole percent fatty acids in hippocampal membranes of adult rhesus macaques (n=9)

|  | **Nuclear** | **Myelin** | **Neuronal** | **Mitochondrial** |
| --- | --- | --- | --- | --- |
| *Total SFA* | 44.10 ± 0.79^a^ | 51.53 ± 1.88^b^ | 49.99 ± 3.01^b^ | 44.37 ± 3.35^a^ |
| *Total MUFA* | 31.02 ± 1.16^a^ | 20.25 ± 0.83^b^ | 22.23 ± 0.69^c^ | 23.49 ± 0.36^d^ |
| *Total PUFAn-6* | 17.18 ± 0.73^a^ | 16.60 ± 1.50^a^ | 16.79 ± 1.92^a^ | 21.38 ± 2.12^b^ |
| 18:2n-6 (LA) | 0.74 ± 0.11^a^ | 1.20 ± 1.75^a^ | 0.82 ± 0.35^a^ | 2.23 ± 0.22^b^ |
| 18:3n-6 (GLA) | 0.09 ± 0.21^a,b^ | 0.09 ± 0.09^a,b^ | 0.06 ± 0.04^a^ | 0.10 ± 0.05^b^ |
| 20:2n-6 | 0.13 ± 0.03^a^ | 0.12 ± 0.10^a^ | 0.12 ± 0.14^a^ | 0.12 ± 0.07^a^ |
| 20:3n-6 (DGLA) | 1.58 ± 0.21^a^ | 0.85 ± 0.17^b^ | 1.01 ± 0.18^b^ | 1.55 ± 0.23^a^ |
| 20:4n-6 (AA) | 8.21 ± 0.52^a,b^ | 8.05 ± 0.41^a^ | 8.69 ± 0.98^b^ | 12.59 ± 1.48^c^ |
| 22:2n-6 | 0.05 ± 0.03^a^ | 0.04 ± 0.09^a,b^ | 0.02 ± 0.02^b^ | 0.02 ± 0.02^b^ |
| 22:4n-6 | 4.97 ± 0.40^a^ | 4.23 ± 0.41^b^ | 4.28 ± 0.62^b^ | 2.89 ± 0.33^c^ |
| 22:5n-6 | 1.41 ± 0.30^a^ | 2.03 ± 0.49^b^ | 1.79 ± 0.27^b^ | 1.88 ± 0.67^a,b^ |
| *Total PUFAn-3* | 7.10 ± 0.73^a^ | 10.97 ± 1.78^b^ | 10.34 ± 0.95^b^ | 10.02 ± 2.04^b^ |
| 18:3n-3 (ALA) | 0.01 ± 0.01^a^ | 0.01 ± 0.02^a^ | 0.01 ± 0.02^a^ | 0.01 ± 0.01^a^ |
| 18:4n-3 (SDA) | 0.01 ± 0.01^a^ | 0.01 ± 0.01^a^ | 0.02 ± 0.03^a^ | 0.02 ± 0.03^a^ |
| 20:5n-3 (EPA) | 1.54 ± 0.77^a^ | 0.54 ± 0.69^b^ | 0.68 ± 0.43^b^ | 0.75 ± 0.92^b^ |
| 22:5n-3 (DPA) | 0.30 ± 0.26^a^ | 0.21 ± 0.17^a^ | 0.17 ± 0.04^a^ | 0.17 ± 0.09^a^ |
| 22:6n-3 (DHA) | 5.25 ± 0.67^a^ | 10.19 ± 1.36^b^ | 9.46 ± 1.07^b^ | 9.06 ± 1.73^b^ |
| *Total Trans* | 0.54 ± 0.48^a^ | 0.56 ± 0.26^a^ | 0.59 ± 0.29^a^ | 0.62 ± 0.31^a^ |

Means with different superscripts across membrane types are significantly different according to Tukey’s HSD test (P<0.05).

SFA: saturated fatty acids, MUFA: monounsaturated fatty acids, PUFAn6: n-6 polyunsaturated fatty acids, LA: linoleic acid, GLA: gamma-linolenic acid, DGLA: dihomo-gamma-linolenic acid, AA: arachidonic acid, PUFAn3: n-3 polyunsaturated fatty acids, ALA: α-linolenic acid, SDA: stearidonic acid, EPA: eicosapentaenoic acid, DPA: docosapentaenoic acid, DHA: docosahexaenoic acid, *trans*: *trans* fatty acids
